# Recurrent Acute Disseminated Encephalomyelitis Presenting as Conus Medullaris Syndrome: A Case Report

**DOI:** 10.3390/medicina60010188

**Published:** 2024-01-22

**Authors:** Dae-Wook Lee, Seok Kang, Nackhwan Kim

**Affiliations:** Department of Physical Medicine and Rehabilitaiton, Korea University Guro Hospital, Seoul 08308, Republic of Korea; daewoogi95@korea.ac.kr (D.-W.L.); caprock@paran.com (S.K.)

**Keywords:** acute disseminated encephalomyelitis, conus medullaris syndrome, magnetic resonance imaging

## Abstract

Acute disseminated encephalomyelitis (ADEM) is an inflammatory demyelinating disorder that typically follows an infection or recent vaccination. Symptoms such as encephalopathy and focal neurological deficits appear weeks after the initial illness, leading to swift and progressive neurological decline. While ADEM in the brain has been well documented, reports of ADEM, specifically in the spinal cord, are relatively limited. A 58-year-old male presented with rapidly progressive bilateral lower extremity tingling, numbness, and mild gait disturbance approximately two days prior to visiting the emergency room. Spinal magnetic resonance imaging revealed a diffuse, longitudinal, high-signal lesion with mild enlargement of the conus and proximal cauda equina. The lesions were predominantly localized in the distal conus and cauda equina, and serial electrodiagnostic studies showed that the lesions progressed toward the proximal conus in tandem with symptom evolution and lacked clear lateralization. The patient was subsequently treated with high-dose steroids for seven days (intravenous methylprednisolone, 1 mg/kg). The patient’s lower extremity weakness gradually improved and he was able to walk independently under supervision three weeks after symptom onset. In this case of spinal ADEM in a middle-aged adult, high-dose steroid treatment led to outstanding neurological recovery from both the initial occurrence and subsequent attacks.

## 1. Introduction

Acute disseminated encephalomyelitis (ADEM) is a rare inflammatory disorder of the central nervous system (CNS) that mainly affects children and young adults [1]. It involves widespread inflammation in the brain and spinal cord and triggers neurological symptoms that often arise quickly after infection or vaccination [2]. While ADEM in the brain has been well documented, reports of ADEM specifically in the spinal cord are relatively limited. Several case reports have highlighted the distinct features of ADEM involving the spinal cord [3,4]. These reports describe a wide range of symptoms, including motor weakness, sensory deficits, bladder dysfunction, and gait disturbances. Notably, the clinical presentation in these cases often resembles that of other spinal cord disorders, such as transverse myelitis or spinal cord demyelination. Case reports have underscored the diagnostic challenges encountered in identifying ADEM in the spinal cord. Owing to the rarity of this condition and its overlapping symptoms with those of other spinal cord disorders, an accurate and timely diagnosis can be challenging. Neuroimaging, particularly spinal cord magnetic resonance imaging (MRI), plays a crucial role in differentiating ADEM from other conditions by revealing multifocal lesions and inflammation in the spinal cord.

Our report focuses on a middle-aged patient experiencing recurrent ADEM, in which symptoms were confined to the spinal cord, specifically manifesting as conus medullaris syndrome.

## 2. Case Presentation

A 58-year-old male presented with rapidly progressive bilateral lower extremity tingling, numbness, and mild gait disturbance approximately two days prior to visiting the emergency room. He reported no history of trauma, drug allergies, or familial diseases. He had been on antihypertensive medication for approximately ten years, had a history of antispasmodic medication four weeks earlier because of diarrhea of unknown origin, and complained of fatigue and lethargy for a few weeks. The initial examination revealed conus medullaris syndrome: saddle hypoesthesia including both L5 and S1 dermatomes, weak anal sphincter motor plus fair motor grade of ankle plantarflexors, loss of anal and bulbocavernosus reflexes, hyperactive deep tendon reflex on both knee jerks, a positive Babinski reflex, and urinary retention. Fundus oculi were normal. Laboratory tests showed increased white blood cell count (13.6 × 10^3^ cells/μL; reference: <11.0 × 10^3^ cells/μL) and mildly elevated C-reactive protein (8.0 mg/L; reference: <5 mg/L). Cerebrospinal fluid (CSF) examination four days after symptom onset revealed 65 mg/dL protein, normal cell counts, and an IgG index of 0.353, without evidence of oligoclonal bands. Tests for hepatitis, syphilis, herpes simplex virus, Epstein–Barr virus, varicella zoster virus, cytomegalovirus, human immunodeficiency virus, antistreptolysin O, mycoplasma, antinuclear antibody, rheumatoid factor, anti-cyclic citrullinated peptide antibody, and HLA-B27 were negative. Vitamin B12 levels and thyroid function test results were within normal ranges.

**Figure 1:**
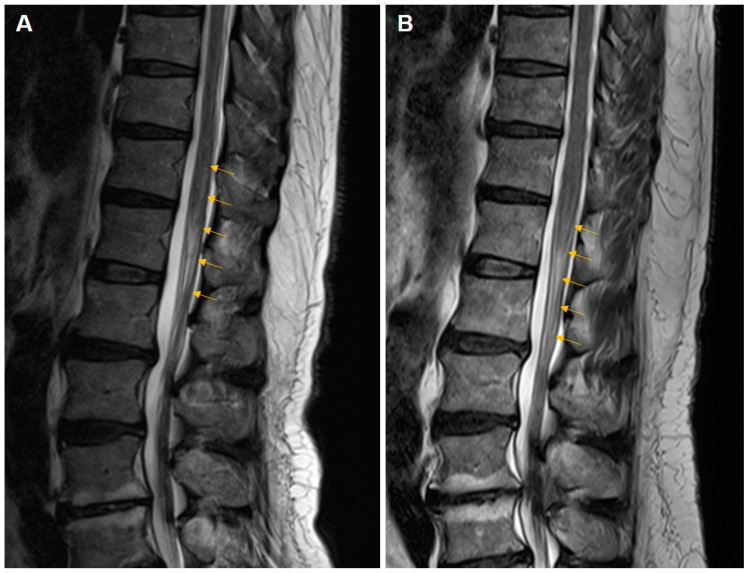
Sagittal T2-weighted views from thoracolumbar spinal magnetic resonance imaging (MRI). MRI at the first attack showed a diffuse longitudinal high-signal lesion (yellow arrows) with mild enlargement of the spinal cord from T12 to L1 (**A**). MRI at the recurrent attack revealed a similar lesion from L1 to L2 (**B**).

Thoracolumbar spinal MRI taken on the fourth day of symptom onset showed a diffuse, longitudinal, high-signal lesion with mild enlargement of the spinal cord from T12 to L1 (Figure 1A). Subtle enhancement was observed in the lesions following administration of gadolinium–diethyltriaminepentaacetic acid. T2-weighted and fluid-attenuated inversion recovery (FLAIR) images showed hyperintense lesions and subtle enhancement in the conus medullaris and proximal cauda equina.

An electrophysiological study 16 days after the onset of symptoms showed delayed latency of F-waves in left peroneal motor stimulation, no response of bilateral H-reflexes, and abnormal spontaneous activities plus polyphasic motor unit action potentials (MUAPs) with reduced recruitment patterns on the left S1–S4 and right S2–S4 myotomes. A somatosensory-evoked potential study showed delayed latencies on bilateral L5 and S1 dermatome stimulation. The patient was treated with high-dose steroids for seven days (intravenous methylprednisolone, 1 mg/kg). His lower extremity weakness gradually improved, and he could walk independently under supervision three weeks after symptom onset. Urinary retention also improved, precluding the need for intermittent catheterization. At the 1-week follow-up, an electrophysiological study revealed delayed latency or no response of F-waves in both peroneal motor stimulations. Abnormal spontaneous activity was localized in the left L5 and bilateral S1 myotomes, and abnormal MUAPs were present in the right S1–S4 and left L5–S4 myotomes. The somatosensory-evoked potential study showed more delayed latencies or no response to bilateral L5 and S1 dermatome stimulation. An electrophysiological study conducted 92 days after symptom onset showed no specific interval change compared to the previous study (Table 1). Mild saddle hypoesthesia lasted for six months after onset.

Eighteen months after the first onset, he visited the outpatient clinic for bilateral lower extremity tingling, numbness, and weakness, which were reportedly similar to the previous symptoms. Significant infection-related symptoms were not specific within three months before the second attack. Sensory deficits were the main complaint and were most severe in the left L5 dermatome. Upper motor neuron signs were observed in both the lower extremities. There were no specific abnormalities on blood tests other than a slightly increased white blood cell count (12.3 × 10^3^ cells/μL). The IgG index was 0.591. Needle electromyography revealed polyphasic and large-amplitude MUAPs with reduced recruitment patterns on the left L5 and bilateral S1 myotomes and abnormal spontaneous activities on the left L5 myotome. MRI at the time of the relapse revealed a similar lesion at the level from L1 to L2 (Figure 1B).

The patient was managed with high-dose steroids for seven days (intravenous methylprednisolone, 1 mg/kg), with the same prescription as the first. The symptoms and signs completely improved two weeks after onset. No similar symptoms occurred during the 3-year follow-up period after the second attack.

## 3. Discussion

In this report, we outline a clinically intricate instance of ADEM that primarily affected the spinal cord, but exhibited concurrent involvement of certain spinal nerve roots. Our assessment attributed this condition to spinal ADEM. During the 3-week progressive phase of the disease, the patient exhibited partial electrophysiological regression following immunosuppressive treatment within one week of symptom onset. A subsequent follow-up 18 months after disease onset revealed a relapse in symptomatology. Serial electrodiagnostic studies enabled the estimation of spatiotemporal changes in lesion progression. The lesions were predominantly localized in the distal conus and cauda equina, progressed toward the proximal conus in tandem with symptom evolution, and lacked clear lateralization. Primarily, demyelination-related impairments prevail and are occasionally accompanied by axonal damage in severe cases. Evident alterations in the S1 dermatomyotome include both latency delays and amplitude decreases. Notably, there was a latency delay in the subsequently affected L5 dermatomyotome; however, no substantial decrease in amplitude was observed. The MRI findings were consistent with the electrodiagnostic outcomes, demonstrating more proximal signal alterations during the initial episode and showing changes below one vertebral level during the relapse. The precise nature of these signals, and whether they reflect residual lesions from the initial inflammation or new manifestations due to recurrence, remains uncertain. Notably, the electrodiagnostic test revealed no myotomal expansion or increased abundance of abnormal spontaneous activity.

ADEM is an immune-mediated disorder that predominantly affects the white matter of the brain and affects both the white and gray matter within the spinal cord [5]. However, our understanding of cases in which ADEM affects the spinal cord alone without concurrent brain involvement remains limited. ADEM exclusively restricted to the brainstem or solely affecting the spinal cord has been reported [6]. Still, descriptions specifically limited to the spinal cord in a single patient are rare. Currently, the primary diagnostic tools for identifying ADEM are MRI and CSF analyses [7]. The key features of spinal ADEM include the abrupt onset of various spinal symptoms; gadolinium enhancement evident across the entire spinal cord in MRI; the presence of pleocytosis in the CSF; and, possibly, a temporary increase in the IgG index within the CSF. ADEM, present in both the cerebral and spinal forms, is frequently observed in children post-infection or post-vaccination [8]. Its occurrence in adults is less frequent, likely due to the concurrence of encephalopathic signs, potentially leading to the under-recognition of spinal symptoms [9]. In a previous study, approximately 16% of patients with cerebral ADEM displayed spinal cord involvement [10]. However, the prevalence of concurrent spinal ADEM may have been underestimated due to the previously discussed factors. MRI of the spinal cord may reveal the confluence of intramedullary lesions featuring multiple regions of heightened signal intensity. Lesions associated with ADEM can manifest simultaneously in both the brain and spinal cord, exclusively within the brain, or solely in the spinal cord [11]. The simultaneous enhancement of lesions in both the brain and spinal cord is often seen as a hallmark feature associated with multiple sclerosis rather than ADEM. Several factors contribute to the overall underdiagnosis of ADEM, particularly spinal ADEM. There are cases of ADEM not compromising the blood–brain barrier, or demonstrating an unconventional enhancement pattern [5]. In complex cases where neurological deficits stem from combined spinal and cerebral involvement, clinicians may inadvertently overlook the spinal aspect of ADEM by solely focusing on brain MRI and not considering the spinal cord. This could lead to an underdiagnosis of the spinal component of the condition [12]. A considerable proportion (approximately 30%) of cases diagnosed with acute encephalitis constitute cerebral ADEM [11], but these cases are rarely subjected to spinal imaging.

Typically, the progression of ADEM follows a monophasic pattern [9] characterized by a clinically progressive phase spanning a few hours to several months. Nonetheless, there have been documented instances of recurrent ADEM, which, in certain cases, may prompt a re-evaluation of the diagnosis, leading to its reclassification as multiple sclerosis [13]. The identification of oligoclonal bands or a sustained elevation in the IgG index within the CSF serves as an indicative factor for the diagnosis of multiple sclerosis rather than ADEM. Although ADEM may exhibit brain imaging features similar to multiple sclerosis, in typical cases of ADEM, diffuse white matter lesions are characterized by less distinct boundaries and are asymmetrically distributed across both the supratentorial and infratentorial regions. Recurrent episodes of ADEM also raise the possibility of myelin oligodendrocyte glycoprotein (MOG) antibody-associated disease. Recent studies have suggested an association between positivity of the anti-MOG antibody and the recurrence rate of ADEM [2,14]. Unfortunately, the anti-MOG antibody was not tested in the present case.

Abnormal spontaneous activities in electromyography of the present case indicate simultaneous peripheral nerve involvement, specifically radiculopathy, which occurs in approximately 82% of ADEM cases [10,15]. ADEM can evolve over a span of up to three months [9], exhibiting progressive characteristics and manifestations during this period. In both pediatric and adult ADEM cases, outcomes can differ; however, in most situations, no new lesions are expected on MRI six months after the initial symptom onset [16]. Symptoms might not completely resolve, often resulting in partial alleviation or lingering manifestations despite treatment or the passage of time in ADEM cases [5]. The anticipated outcome of ADEM is contingent on the response to immunomodulatory treatment [6]. High-dose corticosteroids, particularly when combined with plasma exchange, are considered to be the most effective treatments for ADEM [17]. Oral steroid therapy is usually maintained and tapered over several weeks to lower the risk of early recurrence [14]. In cases when an infection is suspected to be the etiology of ADEM, the preferential treatment approach may include intravenous immunoglobulin administration [18].

## 4. Conclusions

Here, we describe the case of a middle-aged adult who experienced a neuroinflammatory condition specifically confined to the spinal cord, with peripheral involvement of the spinal nerve roots to a lesser degree. A detailed investigation and subsequent follow-up confirmed the diagnosis of spinal ADEM, which was distinct from neuroinflammatory conditions such as Guillain–Barré syndrome, chronic inflammatory demyelinating polyneuropathy, neuromyelitis optica, or multiple sclerosis, specifically due to the absence of concurrent brain involvement. A thorough analysis of radiological findings showing isolated inflammation of the spinal cord, along with systematic follow-up after administering immunomodulatory treatment, may reveal that a substantial proportion of patients categorized as transverse myelitis might actually be affected by spinal ADEM. Further studies are warranted in order to better understand the morbidity, mortality, and long-term outcomes of spinal ADEM.

## Figures and Tables

**Table 1:** Key findings from serial electrodiagnostic studies.

Electrodiagnostic Variables	Side of Extremity	Number of Days after the Onset of Weakness		
		16 Days	23 Days	92 Days
Tibial CMAP (mV)	Right	6.5	6.8	6.3
	Left	6.2	6.4	6.2
Peroneal CMAP (mV)	Right	4.5	3.8	3.9
	Left	4.5	3.2	2.9
Tibial F-wave (msec)	Right	49.2	49.1	49.0
	Left	49.6	49.3	49.1
Peroneal F-wave (msec)	Right	50.1	54.3	52.3
	Left	54.0	NR	58.9
H-reflex of soleus (msec)	Right	NR	NR	NR
	Left	NR	NR	NR
Myotomes revealed ASAs	Right	S2 to S4	S1	S1
	Left	S1 to S4	L5 to S1	L5 to S1
Myotomes revealed abnormal MUAPs	Right	S2 to S4	S1 to S4	S1 to S4
	Left	S1 to S4	L5 to S4	L5 to S4
SSEP stimulated on S1 dermatome (msec)	Right	41.8	45.8	43.5
	Left	41.7	45.6 (s)	48.9 (s)
SSEP stimulated on L5 dermatome (msec)	Right	42.3	48.9	44.2 (s)
	Left	45.4	NR	52.4 (s)

CMAP, compound muscle action potential; ASA, abnormal spontaneous activity; MUAP, motor unit action potential; SSEP, somatosensory-evoked potential; NR, no response; (s), shallow amplitude.

## Data Availability

Data supporting the findings of this study are available upon request from the corresponding author.
